# The Profile of Selected Protein Markers of Senescence in the Placentas of Cows During Early–Mid-Pregnancy and Parturition with and Without the Retention of Fetal Membranes: A Preliminary Study

**DOI:** 10.3390/ijms26125475

**Published:** 2025-06-07

**Authors:** Ewelina Kosztowny, Jacek T. Wawrzykowski, Monika A. Jamioł, Marta Kankofer

**Affiliations:** Department of Biochemistry, Faculty of Veterinary Medicine, University of Life Sciences in Lublin, Akademicka Street 12, 20-033 Lublin, Poland; ewelina.kosztowny@up.lublin.pl (E.K.); jacek.wawrzykowski@up.lublin.pl (J.T.W.); monika.jamiol@up.lublin.pl (M.A.J.)

**Keywords:** placenta, senescence, bovine, pregnancy, parturition, retained fetal membranes

## Abstract

Senescence in placental cells impacts physiological functions and contributes to pathology. Therefore, we examined biochemical markers of cellular senescence—p38, P-p38, p21, and p53—during pregnancy, at parturition, and in cases of retained fetal membranes. Placentomes were collected from pregnant cows (2nd, 4th, and 5th months of gestation) and parturient cows undergoing cesarean section, categorized as NR or RFM. Samples were separated into maternal and fetal parts and analyzed via WB and ELISA. WB confirmed protein presence, while ELISA showed a significant increase in the concentrations of both p38 and P-p38 in the fetal part in the 5th month of gestation as compared to earlier months. No significant differences were observed in the maternal part across pregnancy and parturition. These findings suggest p38 and P-p38 may be important molecules regulating the normal development of the bovine placenta during middle pregnancy. Further studies are needed to elucidate the mechanisms of their action.

## 1. Introduction

Cellular senescence is a process triggered by various physiological signals and internal and external stressors, leading to irreversible cell cycle arrest. One of the best-described factors contributing to senescence is oxidative stress (OS). Reactive oxygen species (ROS) cause DNA damage and activate intracellular signaling cascades, involving certain mediators such as mitogen-activated protein kinases (MAPKs) [1], which include p38. MAPKs are activated via dual phosphorylation of tyrosine and threonine residues [2].

The senescence response is mediated by proteins that inhibit cell cycle progression, particularly cyclin-dependent kinase (CDK) inhibitors such as p21^CDKN1^ and/or p16^INK4/6/CDKN2^, as well as tumor suppressors including p53 and/or retinoblastoma protein pRb [3]. The relative contribution of p53-p21 and p16-pRB pathways to growth arrest may differ depending on the type of stress. Once activated, p53 induces transcriptional activation of p21, promoting cell cycle arrest, while prolonged or severe damage can trigger p16 activation via mitochondrial dysfunction and ROS-mediated p38 MAPK signaling [4].

Senescence pathways are also activated in the placenta [5]. As pregnancy progresses, placental tissue exhibits increasing signs of senescence, reaching a peak near term, which is considered a normal physiological process. However, premature placental senescence has been associated with complications such as premature rupture of fetal membranes and preterm labor in humans [6,7,8]. In fetal membranes (amniotic and chorionic), senescence acts as a signaling mechanism to initiate labor, driven by oxidative stress and the growing metabolic demands of the fetus [9]. Senescence-related placental changes, including activation of the p38 MAPK and upregulation of p21, have been implicated in the onset of parturition [10].

In dairy cows, retained fetal membranes (RFMs) occur when the fetal membranes are not expelled within 8 to 12 h postpartum due to impaired separation of the fetal (cotyledon villi) and maternal (caruncle crypts) parts of the placenta. There are several risk factors for RFM including structural, endocrine, and immunologic changes [11]. Throughout pregnancy, epithelial cells at the feto-maternal interface undergo tightly regulated cycles of proliferation and apoptosis to maintain placental homeostasis. A significant increase in apoptosis occurs toward the end of gestation [12], and RFM can be partially attributed to impaired apoptosis of bovine placental tissues [13].

In complicated pregnancies, evidence points to telomere shortening, reduced telomerase activity, and increased p53-mediated apoptosis [3]. Proteins such as p53 and p21, critical regulators of both apoptosis and senescence, have been identified in human trophoblasts, with altered expression patterns observed in placentas from pathological pregnancies [14,15,16]. Notably, studies suggest that apoptosis and senescence signaling follow distinct, cell-type- and stage-specific patterns in placental tissue [17].

Given the similarities in the structural and inflammatory consequences of both apoptosis and senescence, it is likely that senescence also contributes to placental remodeling and detachment in cows. Disruptions in senescence-related pathways could impair these processes, potentially leading to RFM.

In this study, we hypothesized that cellular senescence contributes to physiological development and maturation of the placenta, required for its attachment at early pregnancy and detachment at parturition, and that disturbances in this process may be involved in the pathology of RFM. Cows with RFM show different biological profiles compared to cows that normally expel fetal membranes. Although the relationship between senescence processes and RFM is unknown, some of the mechanisms, such as effects on the extracellular matrix, trophoblast cell apoptosis, and inflammatory processes, may be relevant to this condition. Can deviations in the senescence process (as in apoptosis) be related to fetal membrane retention?

We propose that placental tissues from cows with RFM show lower concentrations of P-p38 in placentas with RFM, indicating reduced activation of cell senescence signaling compared to that in NR cows. Such deviations in protein activation may disrupt the normal structural and immunological changes necessary for placental separation. This would be consistent with the observed characteristics in cows with RFM, such as reduced concentrations of inflammatory cytokines, impaired apoptosis, reduced expression of adhesion molecules, metabolic disturbances, and abnormal tissue remodeling.

The aim of this study was to verify whether bovine placental tissues undergo p38-dependent senescence by the determination of the profile and concentrations of p38, phosphorylated p38 (P-p38; active form), p21, and p53 at different stages: during early–mid-pregnancy and at parturition with and without the retention of fetal membranes.

## 2. Results

### 2.1. Western Blot

**Figure 1:**
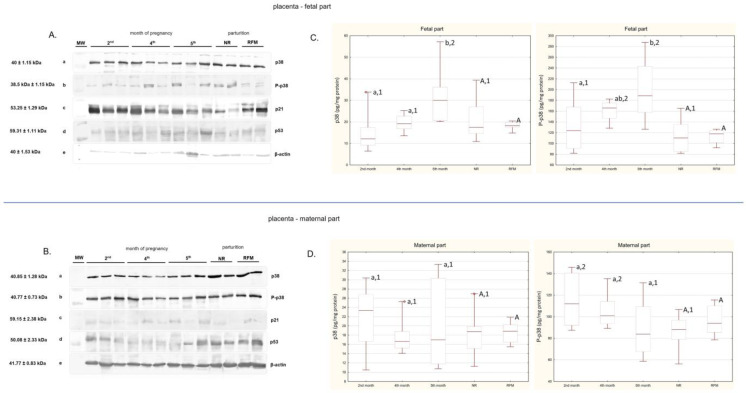
(**A**) Results of Western blotting analysis of proteins p38, P-p38, p21, and p53 (a–d, respectively) in the bovine placenta—fetal part—during pregnancy (2nd, 4th, and 5th months of pregnancy) and at parturition (NR—not-retained fetal membranes; RFM—retained fetal membranes). Blots show three individual representative samples for each gestational month and two samples for each parturition group. β-actin (e) was used as loading control. MW—molecular mass of protein standard (Precision Plus Protein™ Dual Color Standard, Bio-Rad). Original images can be found in the Supplementary Materials, Figure S1A. (**B**) Results of Western blotting analysis of proteins p38, P-p38, p21, and p53 (a–d, respectively) in the bovine placenta—maternal part—during pregnancy (2nd, 4th, and 5th months of pregnancy) and at parturition (NR—not-retained fetal membranes; RFM—retained fetal membranes). Blots show three individual representative samples for each gestational month and two samples for each parturition group. β-actin (e) was used as loading control. MW—molecular mass of protein standard (Precision Plus Protein™ Dual Color Standard, Bio-Rad). Original images can be found in the Supplementary Materials, Figure S1B. (**C**,**D**) p38 (on the left) and P-p38 (on the right) protein concentrations (pg/mg protein) in the bovine placenta (fetal and maternal parts) during pregnancy and at parturition. Data are shown in the box plots, including the minimum and the maximum value (whiskers), the sample median (line in the middle), and the first and third quartiles (frame). Differences (*p* < 0.05) between examined periods of pregnancy (2nd, 4th, and 5th months) are indicated with lowercase letters. Differences (*p* < 0.05) between parturient samples (NR and RFM) are indicated with capital letters. Differences (*p* < 0.05) between samples within individual months of pregnancy and NR are indicated with numbers.

The Western blot results are shown in Figure 1. The presence of the p38 molecule was detected in all examined placental tissue samples during the 2nd, 4th, and 5th months of pregnancy as well as at parturition, with bands corresponding to 40 ± 1.15 kDa in the fetal and 40.85 ± 1.28 kDa in the maternal part. During pregnancy and parturition, bands corresponding to P-p38 of 38.5 kDa ± 1.15 kDa were obtained in the fetal part, which corresponds to the molecular weight of bovine proteins. In the maternal part, the mass of P-p38 was 40.77 ± 0.73 kDa. The bands representing p21 in the fetal and maternal parts had masses of 53.25 ± 1.29 kDa and 59.15 ± 2.38 kDa, respectively. For p53, the signal was located at 59.31 ± 1.11 kDa in the fetal part and 50.08 ± 2.33 kDa in the maternal part.

### 2.2. ELISA Determination of p38 and P-p38 Concentrations

The results of the concentrations of p38 and P-p38 proteins are shown in Figure 1. Quantitative analysis showed that the p38 concentration in the fetal part of the placenta increased significantly (*p* < 0.05) in the 5th month of pregnancy compared to that in the 2nd and 4th months. A similar relationship was observed for fetal P-p38, where its concentration in the 5th month was significantly (*p* < 0.05) higher than in the 2nd month of pregnancy. Both p38 and P-p38 concentrations in the fetal part of the placenta were lower (*p* < 0.05) during physiological parturition (NR) compared to those in the 5th month of pregnancy. There were no significant differences in p38 and P-p38 concentrations between the months studied in the maternal part of the placenta nor between retained and released tissues. P-p38 concentrations in the maternal part of the placenta were lower (*p* < 0.05) during physiological parturition (NR) compared both to those during the 2nd and 4th months of pregnancy. The ratio of P-p38 MAPK to the non-phosphorylated p38 was significantly (*p* < 0.001) elevated at all examined time points.

## 3. Discussion

The placenta serves to protect the fetus throughout pregnancy, but its functional lifespan is limited to the term gestational period. In this study, we used a bovine model to explore whether markers of cellular senescence emerge during pregnancy, at parturition, and in cases of RFM. For this purpose, we examined placental tissues for the presence of p38, its active phosphorylated form P-p38, p21, and p53, and quantified the concentrations of p38 and P-p38 proteins.

Our results show that both p38 and P-p38 were detectable in all pregnancy tissues, with an increase in their concentration in the fetal part during the 5th month compared to that in earlier months. This may suggest that p38 MAPK—a known mediator of cellular stress responses—may be involved in physiological changes during pregnancy. In fact, p38 MAPK activation is important for cell survival in response to DNA damage [18]. However, both p38 and P-p38 concentrations were lower in the fetal part at parturition compared to those in the 5th month, highlighting the necessity for further studies to clarify their role during early pregnancy.

The ruminant placenta is structurally distinct compared to that in other mammals [19]. Parturition in cattle is initiated by a marked increase in fetal cortisol, which stimulates estrogen and PGF_2α_ production, leading to luteolysis and subsequent progesterone withdrawal [20], although these mechanisms do not apply to humans. Available evidence strongly suggests that human labor is regulated by significantly different mechanisms than those found in most animal models [21].

Cellular senescence refers to a state of cell cycle arrest, where cells remain metabolically active and secrete a variety of pro-inflammatory and proteolytic factors, known as the senescence-associated secretory phenotype (SASP). While senescence acts as a tumor-suppressive mechanism, the accumulation of senescent cells over time contributes to chronic inflammation and tissue dysfunction. Different cell types can enter senescence in response to various damage signals, accumulating in tissues and playing both physiological and pathological roles depending on their context [22,23].

To date, no reliable molecular markers for predicting RFM in cattle have been identified. Detecting such markers before delivery remains a significant challenge but would offer valuable insights for early therapeutic intervention. In our previous study focusing on dermatopontin (the representative of cell adhesion proteins), we observed significant differences in its concentration among placental samples collected at parturition, which were later classified retrospectively as retained or non-retained. This observation is clinically important, as it indicates that even at the moment of parturition, cows with RFM exhibit distinct molecular profiles. It suggests that measurable molecular alterations already exist at delivery and opens the possibility that such differences may be detectable even earlier during pregnancy. This provides a rationale for future studies aimed at identifying predictive biomarkers of RFM and its exact time in the pre-parturient period, potentially enabling earlier intervention [24]. Studying cellular metabolism, especially of active proteins, during pregnancy could bring us closer to answering the question of what contributes to this disorder. Although senescence is necessary for normal placental development and fetal growth, aberrant senescence has been linked to various pregnancy-related complications in humans. The current literature lacks sufficient data on protein markers of placental cellular senescence in bovine pregnancy and their relevance to parturition.

Fetal membrane senescence has been studied in mice and humans. Bonney et al. reported a gradual increase in p38 MAPK activation in mouse tissues between gestational days 10 and 18, with the highest levels observed in fetal membranes [25]. Similar findings in human studies [7,10,26] suggest that p38 MAPK mediates senescence in fetal membranes. According to Dixon et al., OS induces cellular senescence and p38 activation, and treatment with NAC (N-acetylcysteine; an antioxidant) suppresses ROS production and p38 activation [27]. In the human placenta, early pregnancy is marked by heightened oxidative stress, accompanied by morphological changes in the uterine arteries and increased expression of antioxidant enzymes [28].

In bovines, Wawrzykowski et al. demonstrated that oxidative stress can accompany placentation [29]. The activity of antioxidative enzymes and total antioxidant capacity (TAC) values decreased in the maternal part, while a slight increase was observed in the fetal part between the 2nd and 3rd months of pregnancy. Concurrently, products of peroxidative damage increased in the maternal part but remained stable in the fetal part. Antioxidant enzyme activity was higher in the 2nd month than in the 3rd. Additionally, increased TAC and lipid peroxidation were observed during placentation compared to those in non-pregnant animals [29]. Comparing early periods of pregnancy (2nd and 3rd months) to parturition, Kankofer et al. suggested that as pregnancy progresses, the imbalance between antioxidant and prooxidant processes tends to increase [30,31].

Jin et al. showed that fetal cells, but not maternal, are susceptible to OS-induced senescence and inflammation [32]. In vitro, fetal membranes exposed to OS activated p38, while untreated controls showed only minimal activation [10]. Similarly, Bonney et al. observed increasing P-p38 concentrations in mouse fetal membranes from day 10 to 18 [25].

During our WB analysis, we observed P-p38 in both the fetal and maternal parts of the placenta. Interestingly, P-p38 concentrations in the fetal part were higher at the 5th month of pregnancy compared to those at the 2nd month, while no significant differences were detected in the maternal part between the studied months. Furthermore, P-p38 concentrations were lower during physiological parturition compared to those during the pregnancy period. The triggering of p38 MAPK-mediated senescence may be dose- and agent-dependent, but this was not assessed in this study. However, the observed increase in p38 and P-p38 proteins in the fetal part of the placenta in the 5th month of pregnancy compared to earlier months may reflect changes related to placental development. Certainly, references to the later months of pregnancy could be helpful in better understanding this mechanism. However, samples older than 5 months were not included in our study due to the difficulties in sample collection. In addition, the ratio of phosphorylated p38 MAPK to the non-phosphorylated p38 was markedly elevated at all examined time points. This suggests that multiple MAPK pathways are active during pregnancy and parturition in cows via upregulation of the active phosphorylated p38 pathway. However, the mechanism leading to this is unknown. It can be assumed that the increase in P-p38 highlights the importance of active signaling mechanisms in the middle of pregnancy. The lack of significant changes in the maternal part may differ in regulatory mechanisms in response to pregnancy progression. Moreover, there were no differences between the RFM and NR groups, which may suggest that other pathways, such as ERK1/2 or JNK, may play a more crucial role, at least at parturition—the moment of sample collection. Further studies in this direction are worth considering.

Generally, the p38 MAPK pathway is described, among others, as a regulator in human trophoblast differentiation [33]. However, there is a lack of data on its pathway in the context of the bovine placenta during pregnancy and parturition. In cattle, it has been linked to hepatocyte apoptosis [34]. Oxidative stress in bovine hepatocytes in vitro triggered phosphorylation and activation of p38 MAPK, which led to increased expression, nuclear translocation, and transcriptional activity of p53 [34]. Madan et al. examined the effects of inhibition of p38 MAPK and extracellular signal-regulated kinase (ERK) signaling on the development of bovine embryos to the blastocyst stage. Individual inhibition of p38 MAPK or ERK signaling did not impair development to the blastocyst stage. However, simultaneous inhibition of both pathways significantly reduced the blastocyst formation, cell number, and immunofluorescence of phosphorylated signaling molecules [35]. Moreover, a study by Doualla-Bell and Koromilas showed that the p38 MAPK pathway was involved in modulating prostaglandin endoperoxide G/H synthase (PGHS-2) isoform expression in bovine myometrial cells [36].

The expected molecular weight of p21, based on its amino acid sequence, is approximately 18–21 kDa, according to the manufacturer. However, our Western blot results showed higher molecular weights (53.25 ± 1.29 kDa in the fetal part and 59.15 ± 2.38 kDa in the maternal part). This discrepancy may be attributed to post-translational modifications. Although transcriptional regulation is considered the primary mechanism controlling p21 expression, there is growing evidence that post-transcriptional and post-translational mechanisms also play significant roles in modulating p21 protein levels and function [37]. Various transcription factors, ubiquitin ligases, and protein kinases modulate the transcription, stability, and cellular localization of p21, thereby affecting its biological activity [38]. Phosphorylation of p21 has been shown to affect both its subcellular distribution [39] and its interaction with other proteins [40].

Research by Menon et al. reported increased p21 expression in human fetal membranes after delivery compared to that in pre-delivery samples [10]. Similarly, a progressive increase in p21 expression was observed with placental maturation in both human [17] and mouse models, where p21 levels were highest on day 18 of gestation across multiple tissue compartments [25].

Although secondary validation techniques such as deglycosylation assays or mass spectrometry were not included in the present study, we acknowledge the potential impact of post-translational modifications in contributing to the observed molecular weight shifts. Additional analyses could provide valuable mechanistic insight and extend the preliminary observations reported here.

Total p53 increased in fetal membrane explants in response to stress induced by cigarette smoke extract [41] with subsequent depletion upon treatment with the antioxidant N-acetylcysteine (NAC). However, phosphorylated p53 (P-p53) was not detected in these samples, suggesting a lack of p53-mediated mechanisms in human fetal membranes [26]. Activation of p53 was not observed in human fetal membranes [10], while mouse fetal membranes showed a gradual increase in p53 activation, with the highest level at day 18 [25].

In our study, we evaluated only the presence of p53; the signal was at 59.31 ± 1.11 kDa in the fetal part and 50.08 ± 2.33 kDa in the maternal part. The limitations of the study are primarily related to the availability of tissue samples. A major limitation of our investigation was the restricted availability of biological material, which prevented the inclusion of ELISA analyses for p53 and p21. This constraint limited our ability to evaluate all markers consistently within the same tissue samples, thereby hindering direct comparisons across molecular targets. Despite these limitations, it is important to acknowledge the critical role of post-translational modifications in regulating p53 activity. Yang et al. demonstrated that O-linked N-acetylglucosamine modification of p53 affects its activity and stability through modulation of phosphorylation and ubiquitination pathways [42]. Moreover, Favetta et al., using primary fetal bovine fibroblasts, observed a gradual decrease in p53 mRNA expression at 20% O_2_. However, p53 protein levels and its phosphorylation at serine 20 increased, suggesting that post-translational modifications may contribute to p53 stabilization during cellular aging [43]. Excessive p53 accumulation, triggered by DNA damage (due to cigarette smoke extract), can initiate apoptosis by compromising mitochondrial membrane integrity, leading to caspase activation. Elevated levels of apoptotic markers in such contexts suggest stress-induced apoptosis in human fetal membranes [41]. In bovine studies, mRNA expression of key apoptotic regulators differed depending on the occurrence of RFM. Apoptotic features were also observed in cells from the maternal part of placentas from cows without RFM, indicating that abnormal apoptosis may only partially contribute to RFM pathogenesis [13].

Boos et al. reported that apoptosis increased as parturition approached, particularly within fetal chorionic epithelial cells. In contrast, placentas associated with RFM exhibited reduced apoptosis, potentially impairing normal tissue turnover and separation processes [12]. Histological assessments of placental membranes in bovine abortions frequently revealed cellular remnants in the chorionic epithelium and adjacent cotyledonary stroma. However, the origin, nature, and mechanistic basis of these findings remain unclear. It is also uncertain whether these changes represent necrosis, apoptosis, or physiological tissue remodeling associated with normal fetal membrane separation during parturition. Currently, the literature addressing these processes remains limited [44].

Understanding the proteins that serve as markers of senescence is crucial to gaining deeper insights into the mechanisms underlying placental development. Further research on these proteins is needed to clarify their role not only in normal placental development during mid-gestation, but also in the pathogenesis of reproductive disorders such as RFM. Linking molecular findings with clinical outcomes could help determine their potential as early biomarkers for RFM risk. Although the current dataset does not yet establish these proteins as clinically usable biomarkers, the observed molecular differences, especially in the fetal part, indicate that p38 MAPKs may serve as candidates for further exploration.

## 4. Materials and Methods

### 4.1. Tissue Collection

Placental tissues were collected from pregnant Holstein-Friesian dairy cows at a local slaughterhouse immediately after slaughter. Tissue dissection was performed under sterile conditions at the same site of the pregnant uterine horn to ensure that placentomes were gathered from comparable locations, minimizing biological variability. Obtained samples covered the early–mid-pregnancy period, specifically between the 2nd and 5th months of gestation (n = 4 for each month), as determined by fetal crown–rump length (CRL), measured from the top of the head (crown) to the base of the tail (rump) using a flexible measuring tape. Based on these measurements, fetuses were assigned to gestational age groups as follows: 8–12 cm for the 2nd month, 19.5–24 cm for the 4th month, and 34–38 cm for the 5th month of pregnancy.

Parturient tissue samples were collected from healthy cows submitted to routine elective cesarean section. Subsequently, the samples were divided into two groups: not-retained fetal membranes (NR; n = 4) and retained fetal membranes (RFM; n = 4). Fetal membranes were considered retained if they were not expelled within 8–12 h after calf removal [45].

### 4.2. Homogenization

Placentomes were carefully separated into maternal caruncles and fetal cotyledons. Individual tissue fragments intended for Western blotting and ELISA were homogenized on ice using Ultra-Turrax T 25 (Ikawerk, Janke and Kunkel Inc., Staufen, Germany) with phosphate buffer (0.05 M, pH = 7.2), Triton X-100. and a protease inhibitor cocktail (Halt™ Protease Inhibitor Cocktail, 87785, Thermo Scientific™, Warszawa, Poland).

For each sample, fragments of tissue measuring approximately 1.8 × 2.7 cm were used. The average mass of the maternal fragments was 3.025 ± 0.123 g, and that of the fetal fragments was 2.102 ± 0.236 g (mean ± SD).

Homogenates were centrifuged at 6500× *g* for 20 min at 4 °C. Supernatants were collected, and the protein concentration was determined using a commercial colorimetric kit (Liquid Cor—Total Protein 60, 2-236, Cormay, Warszawa, Poland). Absorbance measurements were performed in duplicate at 546 nm using a Dr Lange spectrophotometer (Berlin, Germany). Then, samples were frozen at −20 °C for further analysis. Maternal and fetal parts were analyzed separately and individually in duplicate.

### 4.3. Western Blotting (WB) Analysis

Western blotting was conducted to verify the presence of selected proteins in bovine placental tissue. Proteins were separated on polyacrylamide gels using the Laemmli method [46]. The samples were suspended in an SDS reducing buffer (62.5 mM Tris-HCL, pH = 6.8, 20% glycerol, 2% SDS, 5% β-mercaptoethanol, 0.5% bromophenol blue) and denatured at 95 °C for 5 min with shaking.

A 15 µL sample corresponding to 22.5 µg of total protein was loaded into each well. Electrophoresis was performed for 50 min at 200 V using a Mini-PROTEAN^®^ Tetra cell (Bio-Rad, Warszawa, Poland). Following electrophoresis, the proteins were transferred to a PVDF membrane using an Invitrogen™ Blotter (2332037, Thermo Scientific™, Carlsbad, CA, USA) at 100 V for 1 h and 30 min, based on Towbin et al. [47].

Membranes were blocked with Animal-Free Blocker™ 5X Concentrate (SP-5030, Vector Labs, Janki, Poland) in PBS with 0.05% Tween (PBST) for 1 h at room temperature. After blocking and discarding the solution, membranes were incubated overnight at 4 °C with primary antibodies diluted in PBST: p21 monoclonal antibody (1:500, MA5-31479, Thermo Scientific™, Rockford, IL, USA, at least 83% immunogen sequence identity with cow), p53 monoclonal antibody (1:500, MA5-12557, Thermo Scientific™, Rockford, IL, USA), p38 MAPK alpha polyclonal antibody (1:3334, PA5-34769, Thermo Scientific™, Rockford, IL, USA) and phospho-p38 MAPK (Thr180, Tyr182) polyclonal antibody (1:500, 36-8500, Thermo Scientific™, Rockford, IL, USA).

After incubation, membranes were washed 3 times for 10 min each in PBST and then incubated for 2 h at room temperature with secondary antibodies diluted in PBST: goat Anti-Rabbit IgG (1:1333, 31346, Thermo Scientific™, Rockford, IL, USA), or goat Anti-Mouse IgG (1:1333, 31321, Thermo Scientific™, Rockford, IL, USA), conjugated with alkaline phosphatase. After additional washing in PBST (3 times for 10 min each), membranes were incubated with a 1-Step™ NBT/BCIP substrate solution (nitro blue tetrazole chloride and 5-bromo-4-chloro-3-indolyl phosphate in substrate buffer (pH = 9.5, 34042, Thermo Scientific™, Rockford, IL, USA) until band visualization. The reaction was halted with water washing.

β-actin (PA1-183, Thermo Scientific™, Warszawa, Poland) was used as an internal loading control. For pregnancy samples, three individual samples per gestational month were selected for analysis, and for the parturient groups, two samples per group. All analyzed samples represented different individuals and were selected as representative for their respective groups.

The molecular weights of bands were referenced against the Precision Plus Protein™ Dual Color Standard (1610374, Bio-Rad, Warszawa, Poland). Dried membranes were scanned using a GS-710 Calibrated Imaging Densitometer (BioRad, Warszawa, Poland). Signal intensities were analyzed using Image Lab Software 4.0 (Bio-Rad, Warszawa, Poland), and signal intensity was normalized to β-actin.

### 4.4. ELISA

The quantification of proteins p38 and P-p38 in homogenates was determined using enzyme-linked immunosorbent assays: bovine p38 mitogen-activated protein kinase (p38 MAPK) ELISA kit (MBS743801, MyBioSource, San Diego, CA, USA) and bovine phosphorylation p38 mitogen-activated protein kinase (P-p38 MAPK) ELISA kit (MBS747482, MyBioSource, San Diego, CA, USA), according to the manufacturer’s instructions. Each sample was analyzed in duplicate. Absorbance at 450 nm was measured spectrophotometrically using a microplate reader (Labsystem Multiskan RC, Warszawa, Poland). Data analysis, including standard curve generation, was carried out with Genesis software (GENESIS LITE Version 3.03, Life Sciences). The detection limit for p38 was 0.1 ng/mL. The intra-assay variability was less than 10%, while the inter-assay variability was under 12%. Results were reported as picograms per milligram of total protein (pg/mg protein).

### 4.5. Statistical Analysis

The data were analyzed using the Statistica software (Version 13.0, StatSoft, Poland, TIBCO Software Inc., Palo Alto, CA, USA). The normality of the data was calculated through the Shapiro–Wilk test [48]. The homogeneity of variance was assessed using Levene’s test [49]. The non-parametric Kruskal–Wallis test, Mann–Whitney U-test, or Welch’s test was used to determine significant differences between examined groups [50,51,52]. Post hoc power analysis was performed for significant comparisons to assess the reliability of the observed effects. Correction for multiple comparisons was not applied, as the comparisons were predefined and aligned with the experimental design [53]. All data are presented as median ± quartiles, with significance set at *p* < 0.05. Placental tissues (maternal and fetal parts) are compared separately.

## 5. Conclusions

Active p38 mitogen-activated kinase (MAPK) is present in the placenta of cows during both pregnancy and parturition, emphasizing the presence of active signaling mechanisms throughout gestation. Both P-p38 and total p38 may play crucial roles in placental development and function, particularly during middle pregnancy. However, the specific impact of p38 MAPK signaling within the distinct cellular layers of the bovine placenta remains unclear.

Fetal membrane senescence plays a significant role in the mechanisms that regulate delivery at term in humans. Characterizing senescence markers in the bovine placenta could enhance our understanding of the molecular pathways underlying placental health and dysfunction. Such knowledge may ultimately contribute to improved strategies for managing and treating reproductive disorders in livestock.

Investigating senescence markers alongside established apoptotic markers may offer novel insights into the preparation of the bovine placenta for parturition, especially in relation to hormonal regulation and inflammatory signaling. Although senescence has been implicated in parturition in humans and mice, the mechanisms in cattle may differ due to species-specific features such as placental structure and the comparatively greater role of endocrine signals.

## Data Availability

Raw data including WB membranes and ELISA results are available by the first author on request.

## Supplementary Materials

The following supporting information can be downloaded at: https://www.mdpi.com/article/10.3390/ijms26125475/s1.

## Abbreviations

The following abbreviations are used in this manuscript:

RFM	Retained fetal membranes/retention of fetal membranes
NR	Not-retained fetal membranes
OS	Oxidative stress
MAPK	Mitogen-activated protein kinase
P-p38	Phosphorylated p38
