# Supplementary material for: The Profile of Selected Protein Markers of Senescence in the Placentas of Cows During Early–Mid-Pregnancy and Parturition with and Without the Retention of Fetal Membranes: A Preliminary Study
Source: Int J Mol Sci. 2025 Jun 7;26(12):5475. doi: 10.3390/ijms26125475 (PMC12193166; doi:10.3390/ijms26125475)
Supplement: Supplementary file 1 [file ijms-26-05475-s001.zip › Raw data ELISA.pdf]

Maternal part	Sample	Concentrations of p38 pg/mg	Concentrations of P-p38 pg/mg
Group 1 (2nd month of pregnancy)	M13_M	10,5270639	94,49244716
	M13_M	10,43463172	97,52894321
	K21_M	26,06177667	87,30962721
	K21_M	30,38423723	89,53126311
	M19_M	23,60711842	143,7129708
	M19_M	22,77545716	145,9280592
	K2_M	27,57796371	137,2805946
	K2_M	23,06418756	126,5702817
Group 2 (4th month of pregnancy)	M15_M	16,76706758	99,47993801
	M15_M	16,5418563	104,6807272
	K3_M	18,78533058	123,7383844
	K3_M	25,27939424	135,2240314
	M18_M	15,51963293	94,83301724
	M18_M	14,0599557	89,21202159
	K8_M	18,78791788	91,98506579
	K8_M	14,92053869	102,3344166
Group 3 (5th month of pregnancy)	M10_M	30,2950611	89,95454616
	M10_M	33,32247665	84,39524906
	M20_M	11,69771125	68,91587715
	M20_M	11,79439553	83,05580281
	M12_M	19,54276437	131,4435752
	M12_M	30,40908446	128,7695227
	M17_M	14,4691212	58,58751506
	M17_M	10,72056372	66,20352437
Group 4 (not retained) NR	584_M	15,10215994	97,01517003
	584_M	15,47973204	87,98868182
	319_M	18,73681598	78,94279056
	319_M	19,84811781	87,85526066
	1025_M	11,22624792	59,01653915
	1025_M	11,66358002	56,19385211
	48_M	21,40481303	96,86185368
	48_M	26,96875704	106,6830855
	1026_M	18,74727381	93,61198137
Group 5 (retained fetal membranes) RFM	590_M	19,2317547	109,0673076
	590_M	16,79926189	96,3634898
	1027_M	15,4855022	85,4590638
	1027_M	21,25958343	85,62644665
	317_M	18,96923513	78,55788914
	317_M	16,10885538	91,30780225
	50_M	21,88891128	111,1706111
	50_M	18,73089256	115,5605412

Fetal part	Sample	Concentrations of p38 pg/mg	Concentrations of P-p38 pg/mg
Group 1 (2nd month of pregnancy)	M13_F	6,394107794	81,76287423
	M13_F	7,758674704	91,76426608
	K21_F	12,74935664	89,33064175
	K21_F	13,03740853	92,78234712
	M19_F	21,87267153	179,132193
	M19_F	33,81507973	212,6219306
	K2_F	10,5179391	154,6409543
	K2_F	11,58261913	156,1744243
Group 2 (4th month of pregnancy)	M15_F	25,2503533	163,8279542
	M15_F	16,38081802	167,3818633
	K3_F	13,5080892	182,5453086
	K3_F	18,78628493	181,5446222
	M18_F	17,18648266	128,1843748
	M18_F	20,44150931	137,4031813
	K8_F	24,95558824	168,3938118
	K8_F	19,4149682	156,3841445
Group 3 (5th month of pregnancy)	M10_F	31,38657198	202,5417718
	M10_F	30,91042578	193,3628482
	M20_F	20,79145506	126,5615667
	M20_F	29,08608648	138,8585685
	M12_F	40,78924734	283,9683854
	M12_F	57,19623842	287,8558112
	M17_F	20,5187119	176,9966253
	M17_F	20,1126112	183,8435049
Group 4 (not retained) NR	584_F	15,39440042	130,4554638
	584_F	16,2882952	134,4191912
	319_F	10,89632285	81,62783659
	319_F	13,76911939	84,88708387
	1025_F	19,17043593	89,38194406
	1025_F	18,658909	85,10754516
	48_F	34,81948344	165,3518971
	48_F	39,32585407	135,7891945
	1026_F	17,473602	109,9187039
Group 5 (retained fetal membranes) RFM	590_F	18,61129933	100,7453211
	590_F	17,37407349	91,76586307
	1027_F	20,03695782	126,4182791
	1027_F	19,50230938	123,080747
	317_F	17,94957974	117,3889531
	317_F	17,78151699	118,4379299
	50_F	14,81716531	101,653321
	50_F	20,4019319	124,7252304
