# Supplementary material for: The Profile of Selected Protein Markers of Senescence in the Placentas of Cows During Early–Mid-Pregnancy and Parturition with and Without the Retention of Fetal Membranes: A Preliminary Study
Source: Int J Mol Sci. 2025 Jun 7;26(12):5475. doi: 10.3390/ijms26125475 (PMC12193166; doi:10.3390/ijms26125475)
Supplement: Supplementary file 1 [file ijms-26-05475-s001.zip › WB normalised to beta-actin.pdf]

FETAL										
Term	lane	B-actin	p21	p21 norm	p38	p38 norm	p38 P	p38 P norm	p53	p53 norm
2nd	2	248	11138	44,9	3140	12,7	265	1,1	1010	4,1
2nd	3	254	7913	31,2	2780	10,9	615	2,4	861	3,4
2nd	4	497	8819	17,7	3732	7,5	264	0,5	1144	2,3
4th	5	175	11185	63,9	3583	20,5	399	2,3	1097	6,3
4th	6	321	6511	20,3	1955	6,1	1803	5,6	846	2,6
4th	7	665	4831	7,3	1760	2,6	300	0,5	1978	3,0
5th	8	1454	7085	4,9	2540	1,7	1877	1,3	1215	0,8
5th	9	344	4575	13,3	2661	7,7	126	0,4	1444	4,2
5th	10	475	4632	9,8	4107	8,6	1320	2,8	3494	7,4
NR	11	501	3178	6,3	4123	8,2	1381	2,8	1182	2,4
NR	12	306	1447	4,7	3268	10,7	2199	7,2	1169	3,8
RFM	13	532	5812	10,9	3231	6,1	607	1,1	1803	3,4
RFM	14	609	9260	15,2	3277	5,4	478	0,8	1705	2,8

MATERNAL										
Term	lane	B-actin	p21	p21 norm	p38	p38 norm	p38 P	p38 P norm	p53	p53 norm
2nd	2	1563	536	0,3	3704	2,4	3948	2,5	4715	3,0
2nd	3	1703	535	0,3	2980	1,7	3792	2,2	2885	1,7
2nd	4	1309	276	0,2	3431	2,6	5304	4,1	2594	2,0
4th	5	1535	194	0,1	2805	1,8	8280	5,4	1504	1,0
4th	6	1301	1010	0,8	2150	1,7	3139	2,4	1788	1,4
4th	7	447	337	0,8	1583	3,5	2641	5,9	2004	4,5
5th	8	1837	641	0,3	2997	1,6	2813	1,5	1366	0,7
5th	9	1842	313	0,2	3321	1,8	3422	1,9	1824	1,0
5th	10	1495	1073	0,7	4616	3,1	4485	3,0	5338	3,6
NR	11	1919	500	0,3	6740	3,5	4223	2,2	4965	2,6
NR	12	2148	84	0,04	4364	2,0	3921	1,8	3503	1,6
RFM	13	2417	882	0,4	5817	2,4	4003	1,7	3999	1,7
RFM	14	2557	286	0,1	4654	1,8	5192	2,0	6231	2,4
