# Supplementary figures and images for: The Profile of Selected Protein Markers of Senescence in the Placentas of Cows During Early–Mid-Pregnancy and Parturition with and Without the Retention of Fetal Membranes: A Preliminary Study
Source: Int J Mol Sci. 2025 Jun 7;26(12):5475. doi: 10.3390/ijms26125475 (PMC12193166; doi:10.3390/ijms26125475)

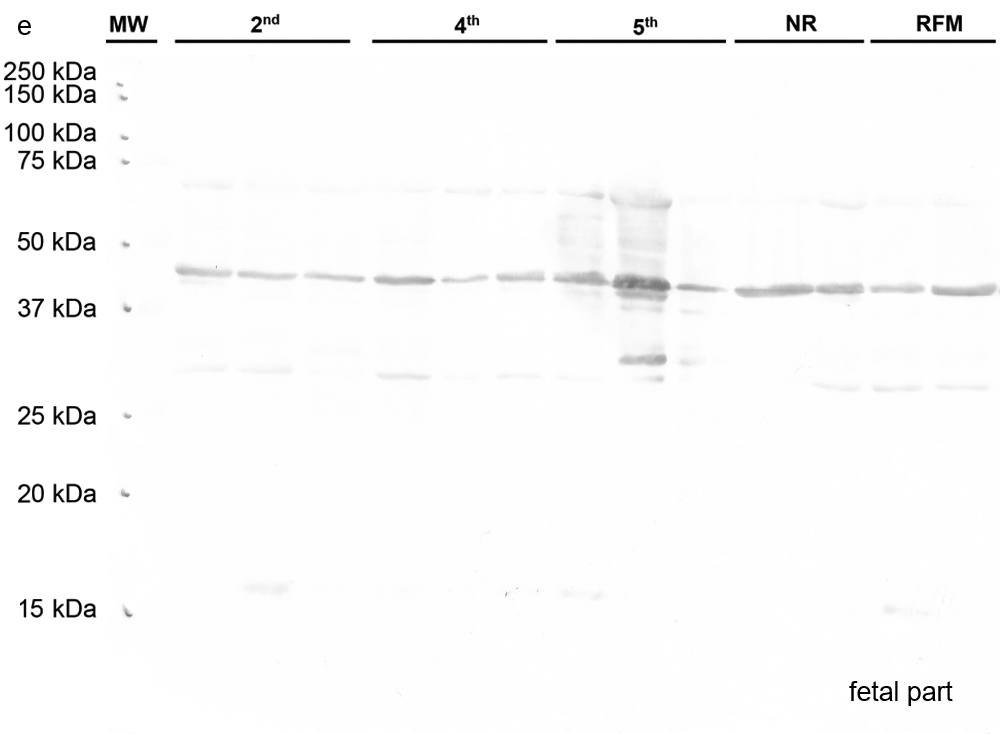

Supplement: Supplementary file 1 [file ijms-26-05475-s001.zip › Figure S1A. Fetal b-actin. Western Blot.tif]

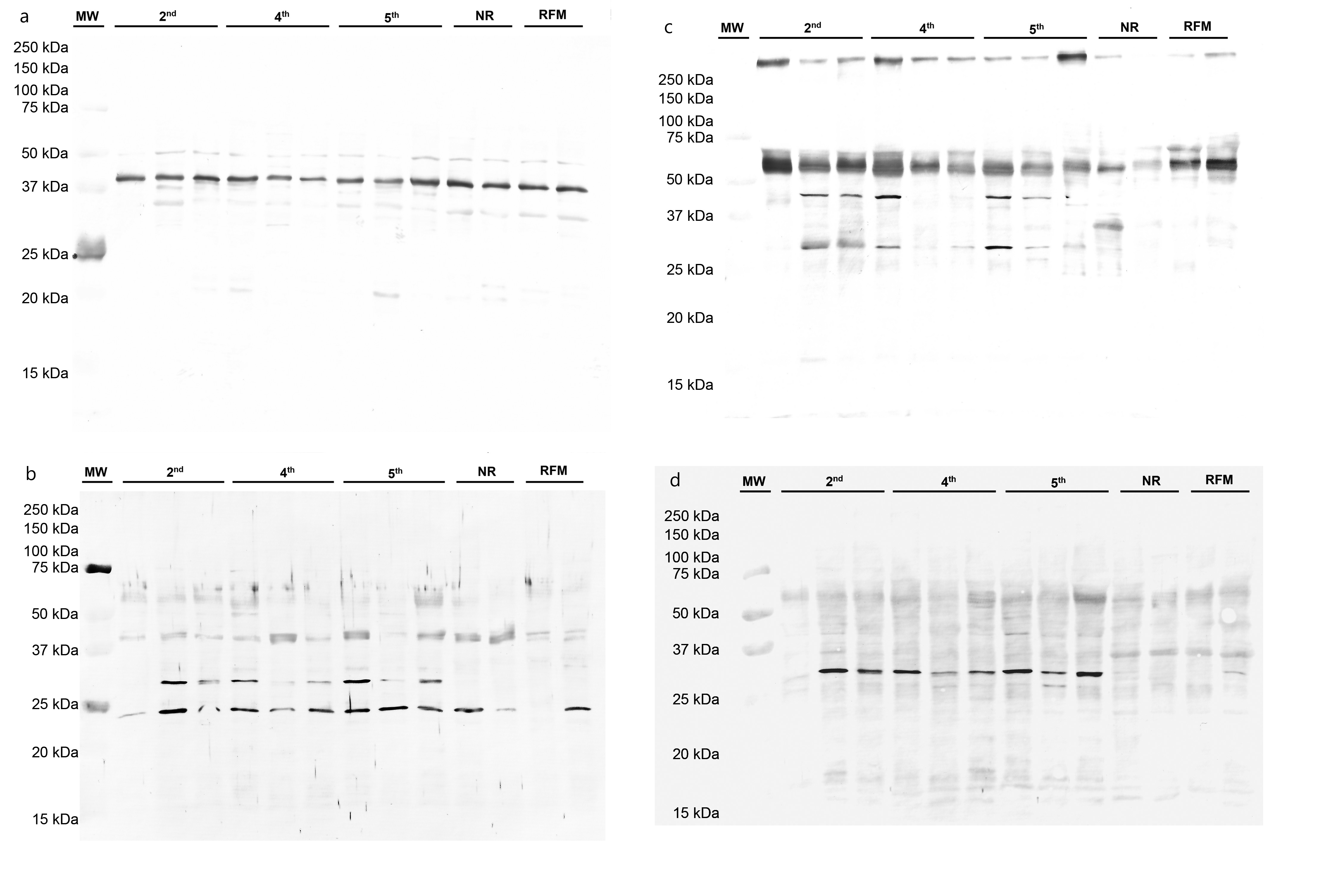

Supplement: Supplementary file 1 [file ijms-26-05475-s001.zip › Figure S1A. Western Blot.tiff]

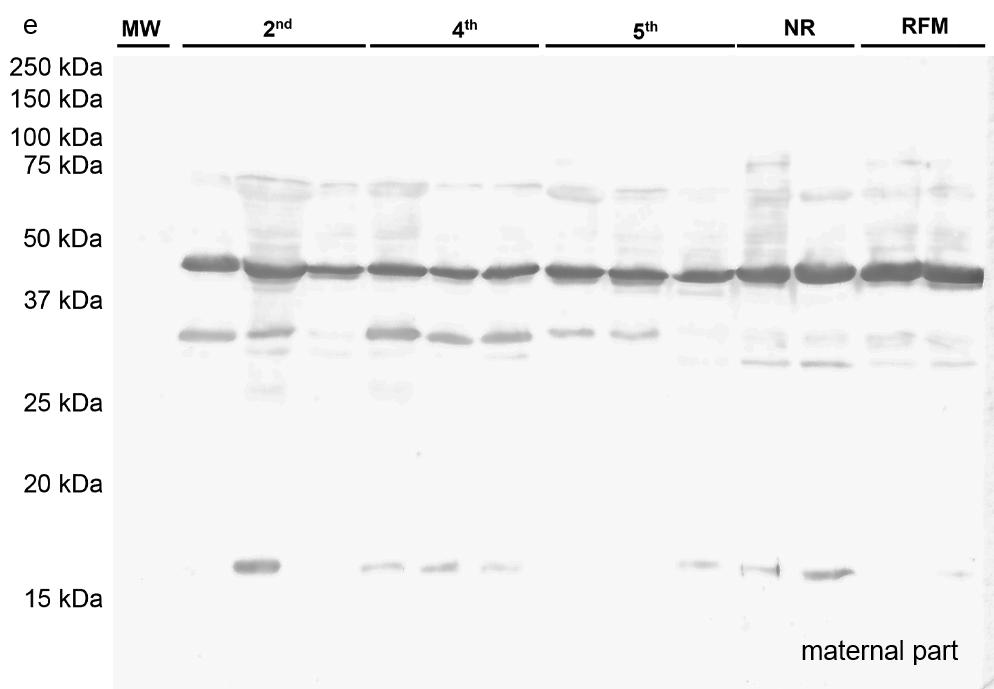

Supplement: Supplementary file 1 [file ijms-26-05475-s001.zip › Figure S1B. Maternal b-actin. Western Blot.tif]

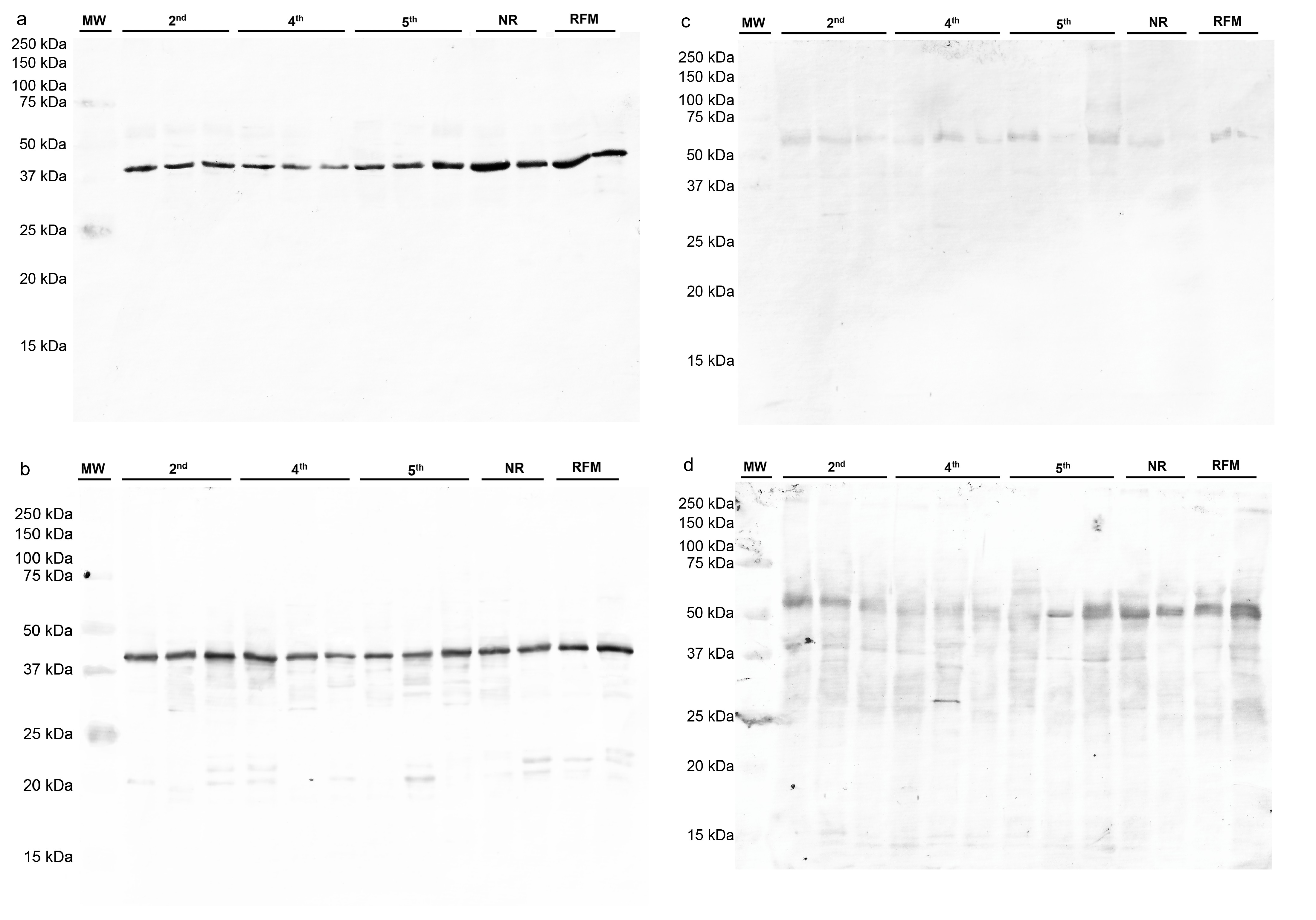

Supplement: Supplementary file 1 [file ijms-26-05475-s001.zip › Figure S1B. Western Blot.tif]
